# The bactericidal activity of glutaraldehyde‐impregnated polyurethane

**DOI:** 10.1002/mbo3.378

**Published:** 2016-06-03

**Authors:** Sandeep K. Sehmi, Elaine Allan, Alexander J. MacRobert, Ivan Parkin

**Affiliations:** ^1^ Department of ChemistryMaterials Chemistry Research CentreUniversity College London20 Gordon StreetLondonWC1H 0AJUnited Kingdom; ^2^ UCL Division of Surgery and Interventional ScienceUniversity College LondonRoyal Free Campus, Rowland Hill StreetLondonNW3 2PFUnited Kingdom; ^3^ Division of Microbial DiseaseUCL Eastman Dental InstituteUniversity College London256 Gray's Inn RoadLondonWC1X 8LDUnited Kingdom

**Keywords:** Antibacterial, biocide, glutaraldehyde, hospital‐acquired infections, polymer

## Abstract

Although glutaraldehyde is known to be bactericidal in solution, its potential use to create novel antibacterial polymers suitable for use in healthcare environments has not been evaluated. Here, novel materials were prepared in which glutaraldehyde was either incorporated into polyurethane using a simple “swell‐encapsulation‐shrink” method (hereafter referred to as “glutaraldehyde‐impregnated polyurethane”), or simply applied to the polymer surface (hereafter referred to as “glutaraldehyde‐coated polyurethane”). The antibacterial activity of glutaraldehyde‐impregnated and glutaraldehyde‐coated polyurethane samples was tested against *Escherichia coli* and *Staphylococcus aureus*. Glutaraldehyde‐impregnated polyurethane resulted in a 99.9% reduction in the numbers of *E. coli* within 2 h and a similar reduction of *S. aureus* within 1 h, whereas only a minimal reduction in bacterial numbers was observed when the biocide was bound to the polymer surface. After 15 days, however, the bactericidal activity of the impregnated material was substantially reduced presumably due to polymerization of glutaraldehyde. Thus, although glutaraldehyde retains antibacterial activity when impregnated into polyurethane, activity is not maintained for extended periods of time. Future work should examine the potential of chemical modification of glutaraldehyde and/or polyurethane to improve the useful lifespan of this novel antibacterial polymer.

## Introduction

The high rate of hospital‐acquired infections (HAIs) and the increasing incidence of antibiotic resistance present a major threat to healthcare worldwide as many infections become increasingly difficult to treat (Jones et al. 2008). Several hospital‐acquired bacterial pathogens have become resistant to multiple antibiotics and this is a major problem for patients undergoing chemotherapy or surgery in whom the ability to treat infection is critical. Despite the presence of strict hygiene protocols in hospitals, transfer of bacteria between healthcare personnel, patients, and the external environment is a critical issue and novel strategies are needed to reduce the environmental reservoir of bacteria associated with HAIs. One approach is the use of self‐sterilizing, antibacterial surfaces on hospital surfaces.

Biocides are extensively used in healthcare for disinfecting surfaces and water, for antisepsis, preserving pharmaceutical products and sterilizing medical devices (Russell et al. 1997) and are extremely effective in controlling HAIs (Maillard 2005).

**Figure 1:**
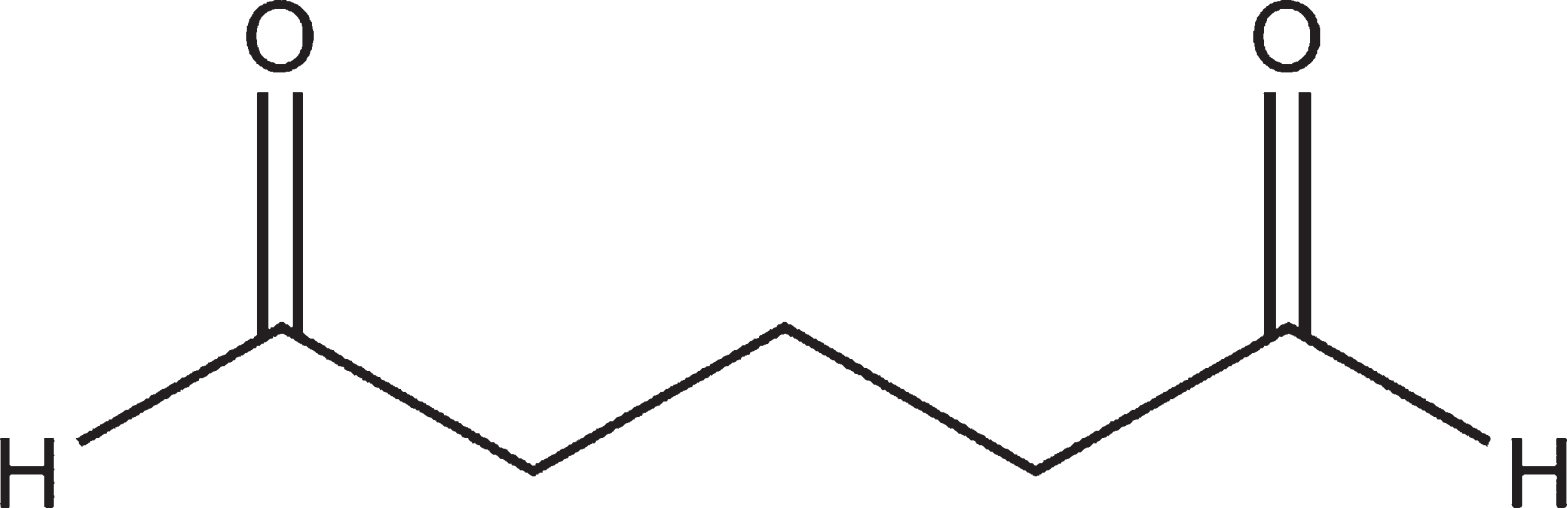
Chemical structure of glutaraldehyde.

Glutaraldehyde is a saturated dialdehyde which has commonly been used as a disinfectant and chemical sterilant in hospitals (Fig. 1). The antimicrobial activity of the biocide is due to the alkylation of hydroxyl, carbonyl and amino groups which affects DNA, RNA and protein synthesis (McGucken and Woodside 1973). Studies have shown strong binding of glutaraldehyde to the outer membrane of *Escherichia coli* and inhibition of membrane transport in other Gram‐negative bacteria, in addition to inhibition of RNA, DNA and protein synthesis (Maillard 2002).

Glutaraldehyde is not active against bacterial cells when in acidic aqueous solutions, however, when activated at pH 7.5–8.5, the solution becomes biocidal. More reactive sites (hydroxyl, carbonyl and amino groups) are formed at the bacterial cell surface at higher pH which leads to a faster bactericidal effect (Rutala and Weber 2004). In vitro studies have reported that alkaline glutaraldehyde solutions are also highly effective against vegetative bacteria including *Staphylococcus aureus*,* E. coli* and *Pseudomonas aeruginosa* in less than 2 min, *Mycobacterium tuberculosis*, fungi, and viruses in under 10 min and spores of *Bacillus* and *Clostridium* species within 3 h (McDonnell and Russell 1999; Rutala and Weber 2004).

One major drawback of alkaline solutions of glutaraldehyde is that they retain activity for only approximately 14 days as the glutaraldehyde molecule begins to polymerize (Migneault et al. 2004). When the biocide polymerizes, the active sites (aldehyde groups) are blocked and biocidal activity is reduced (Kiernan 2000). Polyurethane is a widely used biocompatible polymer and has replaced other common polymer substrates, such as silicone and polyvinyl chloride (PVC) in hospitals as it is more comfortable for patients (McKeen 2014). It is used for surgical prostheses, catheters, and artificial heart, kidney, and blood vessels. Polyurethane elastomers are used in formulating hemostatic coatings and biomedical adhesive systems (Blass 1999). More flexible polyurethane foams are also used in producing bandages, surgical dressings, and absorbent materials for general hospital practice (Ratner 2004).

In this work, we prepared novel materials in which glutaraldehyde was either incorporated into polyurethane using a simple “swell‐encapsulation‐shrink” method (hereafter referred to as “glutaraldehyde‐impregnated polyurethane”), or applied to the polymer surface (hereafter referred to as “glutaraldehyde‐coated polyurethane”). The antibacterial activity of glutaraldehyde‐impregnated and glutaraldehyde‐coated polyurethane samples was tested against *E. coli* and *S. aureus* with exposure times of 2 h. We hypothesized that incorporation of glutaraldehyde into polyurethane may reduce the rate of glutaraldehyde polymerization, thus prolonging the useful lifespan of the biocide. To determine if this was the case, the antibacterial activity of the polymers was retested after 15 days.

## Material and Methods

### Chemicals and substrates

Glutaraldehyde solution grade II (25 wt.% in H_2_O) and acetone were purchased from Sigma‐Aldrich Chemical Co., United Kingdom. In all synthetic work, deionized water was used (resistivity 15 MΩ cm) and the substrate was medical grade flat polyurethane sheets (thickness 0.8 mm) purchased from American Polyfilm Inc. (Branford, CT).

### Material synthesis and characterization

The following samples were prepared for antimicrobial testing: control polyurethane polymer samples (solvent treated only), glutaraldehyde‐impregnated samples (biocidal swelling solution treated), and glutaraldehyde‐coated samples (biocidal solution treated only). The infrared absorbance spectra of the polyurethane polymer samples were measured using a Bruker Platinum ATR, within the range 4000–400 cm^−1^ with an accumulation of 15 scans per sample. X‐ray photoelectron spectroscopy (XPS) analysis of the solvent‐treated and glutaraldehyde‐impregnated polyurethane polymer samples was carried out using a Thermo Scientific, (United Kingdom) *K‐α* spectrometer to classify the different elements present as a function of polymer depth. All binding energies were calibrated to the C 1s peak at 284.5 eV.

### Wetting properties

Equilibrium contact angle measurements (~5.0 *μ*L) were carried out on the solvent‐treated and biocide‐impregnated polymer samples, using an FTA 1000 Drop Shape Instrument. The contact angle measurements for each sample were taken to be the average value over ≥10 measurements, using a droplet of deionized water dispensed by gravity from a gauge 30 needle, with a camera that photographed the samples side on. The data were analyzed using FTA32 software.

### Antimicrobial activity

The antibacterial activity of the following polyurethane samples (1 cm^2^) was tested against *S. aureus* 8325‐4 and *E. coli* ATCC 25922: (1) solvent treated (control), (2) glutaraldehyde‐impregnated and (3) glutaraldehyde‐coated polyurethane. The bacteria were stored at −70°C in Brain–Heart Infusion broth (BHI, Oxoid) containing 20% (v/v) glycerol and propagated on either MacConkey agar (MAC, Oxoid) in the case of *E. coli* or Mannitol Salt agar (MSA, Oxoid) in the case of *S. aureus* for a maximum of two subcultures at intervals of 2 weeks. The protocol used in this investigation (Data S1.) was adapted from that of Noimark et al. (2013) at the Materials Chemistry Research Centre at University College London.

Each experiment contained three technical replicates and the experiment was repeated three times. The statistical significance of the following comparisons was analyzed using the Mann–Whitney *U* test: (1) control (polymer only) versus inoculum; (2) glutaraldehyde‐impregnated or glutaraldehyde‐coated versus control; (3) glutaraldehyde‐impregnated vs glutaraldehyde‐coated polyurethane. To determine the shelf‐life of the glutaraldehyde solution (0.25 wt.% in H_2_O) used in the antibacterial investigation, the antibacterial activity of the samples was determined immediately after preparation and then after 15 and 30 days.

## Results and Discussion

### Material synthesis and characterization

Glutaraldehyde solution grade II (containing 3% w/v glutaraldehyde in water) was purchased from Sigma‐Aldrich Chemical Co. and diluted by a factor of 100 contain only ~0.03% w/v glutaraldehyde. By diluting the solution, the pH increased from ~pH 3.0 to ~pH 8.0. A low concentration of glutaraldehyde was used to minimize potential toxicity to staff, patients and visitors in a clinical setting. To prepare the glutaraldehyde‐impregnated samples, the biocidal solution was prepared in a 1: 1 mix of water and acetone (based on volume) for 24 h at ambient temperature and pressure, washed and towel dried. This method ensures uniform coating and impregnation of the biocide into the polymer substrate and across the surface. To prepare the glutaraldehyde‐coated polymer, the dilution was performed in pure water.

The infrared absorbance spectra of the samples were obtained by ATR (Fig. S1–S3). The spectra did not show any significant changes between the treated and untreated polymer samples in the range analyzed, which can be attributed to the strong absorbance bands of the polymer and due to the low concentrations of glutaraldehyde present in the samples. However, the similarity in the spectra of glutaraldehyde‐impregnated and glutaraldehyde‐coated polymer samples confirmed that the “swell‐encapsulation‐shrink” technique does not affect a chemical change in the polyurethane substrate.

XPS was used to identify the presence of chemical elements in the control, glutaraldehyde‐impregnated and glutaraldehyde‐coated polyurethane to determine the efficacy of the “swell‐encapsulation‐shrink” method. XPS depth profile data showed that the carbon content did not decrease, but the composition of oxygen and nitrogen decreased with polymer depth. This was displayed across all samples including the control polyurethane sample and cannot be attributed to the addition of glutaraldehyde. For all polymer types analyzed, peaks corresponding to the presence of carbon (284.5 eV), oxygen (531.7 eV) and nitrogen (399.3 eV) on the surface were observed, with no significant difference in percentage element composition between the control and biocide‐treated samples (data not shown). In addition, XPS data indicated the presence of a new carbon environment on the surface of both modified polyurethane samples (287.6 eV). This change indicates the presence of a new “ketone‐type” C 1s environment, corresponding to the glutaraldehyde molecule (Fig. S4). Furthermore, another oxygen peak was observed (530.7 eV) exclusively on the surface of the glutaraldehyde‐impregnated polyurethane sample, suggesting the presence of an aldehyde group on the surface, representing glutaraldehyde (Fig. S5).

### Wetting properties

The water contact angles of untreated and treated polyurethane samples indicated that the untreated polymer surface presents a hydrophobic surface (Table 1). The addition of acetone, water, or glutaraldehyde resulted in a negligible change in material hydrophobicity, varying in contact angle by a maximum of +/−1 degree.

**Table 1:** Average contact angle measurements (^o^) ± standard deviation, of water on a range of polyurethane polymer: untreated, solvent‐treated (control), glutaraldehyde‐impregnated, and glutaraldehyde‐coated samples

Polymer sample	Contact angle (^o^) ±standard deviation
Untreated	93 ± 1.3
Control	93 ± 0.9
Glutaraldehyde‐impregnated	93 ± 1.4
Glutaraldehyde‐coated	94 ± 0.7

### Antibacterial activity

**Figure 2:**
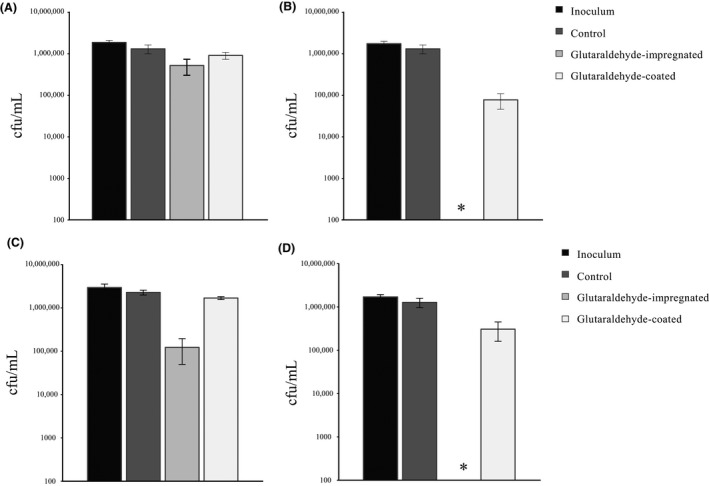
Viable counts of *S. aureus* after incubation at 20°C on modified polyurethane squares for: (A) 30 min and (B) 1 h, and viable counts of *E. coli* after incubation on modified polyurethane squares for: (C) 1 h and (D) 2 h. Control samples are solvent treated. *indicates bacterial numbers reduced below the detection limit of 100 cfu/mL.

The information obtained from XPS data suggested that the biocide mainly resides on the polymer surface even when swell‐encapsulated. The antibacterial activity of glutaraldehyde‐impregnated polyurethane was compared with glutaraldehyde‐coated polyurethane against *E. coli* and *S. aureus* (Fig. 2).

After 30 min of incubation, the control polymer and glutaraldehyde‐coated samples did not show significant kill of *S. aureus*, whereas the glutaraldehyde‐impregnated polyurethane demonstrated an ~0.5 log reduction in bacterial numbers (*P* < 0.001, Fig. 2A). *S. aureus* was also exposed to the samples for 1 h (Fig. 2B); here the control sample did not show any significant kill, but the glutaraldehyde‐coated polyurethane resulted in a ~1.5 log reduction in bacterial numbers. Glutaraldehyde‐impregnated polyurethane exhibited the greatest kill with the numbers of *S. aureus* reduced to below the detection limit of 100 cfu/mL (≥4 log; *P* < 0.001).

Following 1 h of incubation (Fig. 2C), neither the control polyurethane sample, nor glutaraldehyde‐coated polyurethane displayed any significant reduction in the numbers of *S. aureus*. However, the glutaraldehyde‐impregnated polyurethane resulted in a statistically significant reduction in *E. coli* numbers of ~1.3 log (*P* < 0.001). After exposing the *E. coli* to the polymer samples for 2 h (Fig. 2D), the control polyurethane did not display any significant bacterial kill, but the glutaraldehyde‐coated polyurethane produced an ~0.7 log reduction in bacterial numbers (*P* < 0.001). Moreover, glutaraldehyde‐impregnated polyurethane exhibited highly significant bactericidal activity (*P* < 0.001), reducing bacterial numbers to below the detection limit (≥4 log).

The difference in efficacy between the glutaraldehyde‐impregnated polymer and the glutaraldehyde‐coated samples could be due to a difference in the rate of glutaraldehyde polymerization that occurs within the biocidal film during its preparation. It is possible that monomeric glutaraldehyde molecules on the surface readily undergo polymerization, whereas encapsulation of the biocide prevents or delays this process. The XPS data indicated that a low level of biocide was mainly present close to the polymer surface in the glutaraldehyde‐impregnated polyurethane. However, the fact that it showed increased bactericidal activity compared to the glutaraldehyde‐coated polyurethane indicates that even this low level of biocide was sufficient to reduce the numbers of bacteria in contact with the polymer surface. It is possible that the low level of glutaraldehyde contained within the polymer is protected from polymerization and slowly leaches out leading to an increased concentration of the biocide at the polymer surface. In addition, it is possible that a larger proportion of the biocide is lost from the glutaraldehyde‐coated surface compared to the glutaraldehyde‐ impregnated samples when the samples are washed and towel‐dried.

The stability of the glutaraldehyde‐impregnated and glutaraldehyde‐coated polyurethane was investigated by repeating the antibacterial tests after the polymers had been stored for 15 days at room temperature. After this time period, the bactericidal activity displayed by the glutaraldehyde‐impregnated polyurethane after 2 h of exposure decreased dramatically from a ≥4 log to ~0.5 log reduction in bacterial numbers for both *E. coli* and *S. aureus*. Furthermore, there was minimal bactericidal activity shown by the glutaraldehyde‐impregnated and glutaraldehyde‐coated samples tested against *E. coli* after 1 h (Fig. S6). The bactericidal activity of the samples was no longer detectable after 30 days.

**Figure 3:**
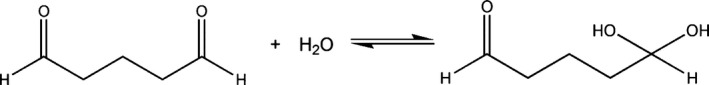
Equilibrium reaction scheme in basic conditions (adapted from Margel and Rembaum (1980)).

Glutaraldehyde molecules undergo polymerization at alkaline pH which blocks the active sites on the biocide and thus reduces its biocidal activity (Aiello et al. 2007). It has been suggested that a plausible mechanism for the polymerization of monomeric glutaraldehyde involves an Aldol condensation reaction where dehydration occurs, yielding ethylene linkages conjugated with aldehyde functions (Migneault et al. 2004). Furthermore, polymerization in basic conditions shifts the equilibrium of the reaction scheme shown in Fig. 3 to the left, so under basic conditions, more free aldehyde groups are available (Margel and Rembaum 1980). This suggests that by increasing the pH of the biocidal solution, we have encouraged the formation of free aldehyde groups which can undergo Aldol condensation reactions to form poly‐glutaraldehyde thus limiting the antimicrobial activity that is observed. Another explanation why the glutaraldehyde impregnated within the polymer displays better antibacterial activity than the surface‐associated molecule is that glutaraldehyde polymerization occurs more slowly within the polymer as a result either of the reduced oxygen concentration (Migneault et al. 2004) or the decreased opportunity for monomer–monomer interaction as a result of reduced diffusion. Presumably it is thus able to continuously leach from the polymer and maintain effective concentrations at the polymer surface.

XPS data implied the possibility of a new chemical environment on the polyurethane surface after treatment with the biocidal solution. The many benefits of using glutaraldehyde as a biocide include its cost effectiveness (D'Ercole et al. 2002) and excellent material compatibility (Anon 2003); however, its major disadvantage is that it has relatively low mycobacterial activity (Stanley 1999). Its biocidal activity is very similar to formaldehyde, acting either by denaturing proteins or by modifying nucleic acids by alkylation. It is favored by an alkaline pH (e.g. 8.0), but in such conditions, its disinfectant activity decreases as the molecule polymerizes (Maris 1995). After 15 days of storage, the antibacterial activity of the samples had significantly reduced. One way to potentially resolve this issue may be to covalently attach glutaraldehyde to the polymer surface to prevent polymerization and increase its stability at higher pHs.

For the first time, we have demonstrated antibacterial activity by glutaraldehyde incorporated into polyurethane square sheets with the potential to be used in the healthcare industry in lowering the risk of spreading nosocomial infections. In contrast to our previous work which focuses on light‐activated materials, these materials showed potent bactericidal activity in the dark (Noimark et al. 2009a,b, 2012, 2013, 2014; Page et al. 2009; Piccirillo et al. 2009; Perni et al. 2010, 2011; Sehmi et al. 2015a,b). Previous studies carried out by Sehmi et al. (2015a,b) demonstrated a greater swelling ability of polyurethane compared to silicone, which is also a widely used polymer in hospital surface and medical device applications. Thus, polyurethane was used in this investigation to ensure a greater concentration of glutaraldehyde was impregnated into the polymer matrix. The results of the current investigation have shown that glutaraldehyde may have potential in reducing the incidence of HAIs, provided we can increase the longevity of its antibacterial activity. The bactericidal activity of glutaraldehyde is retained for longer when impregnated into the polymer, compared to simply coating it onto the surface of the polymeric material, but further work is needed to identify new methods for impregnation which will increase its antibacterial activity over time. To our knowledge, this is the first investigation of a glutaraldehyde‐impregnated polymer as an antimicrobial material.

In summary, we have shown the bactericidal activity of glutaraldehyde‐impregnated and glutaraldehyde‐coated polyurethane against *S. aureus* and *E. coli*. To minimize toxicity, we have used a much lower concentration of glutaraldehyde than that reported in the literature (Gorman and Scott 1979; Bailly et al. 1991; Herruzo‐Cabrera et al. 1999; Lerones et al. 2004; Li et al. 2013), Functional tests were carried out to investigate the hydrophobicity of the polyurethane surface, as well as the stability of the biocide. The results showed that the wetting properties of the polyurethane did not change as a result of adding glutaraldehyde or when the samples were left for a 30‐day period (Table S1), which is important because a hydrophobic surface can reduce biofilm formation on the polymer surface. With glutaraldehyde‐impregnated polymer, the numbers of *S. aureus* and *E. coli* were reduced by >4 log after 1 or 2 h of exposure, respectively. The outstanding results obtained from using the “swell‐encapsulation‐shrink” technique to prepare the polymer are beneficial in dark or poorly lit environments which are often found in older hospital wards in UK hospitals. Glutaraldehyde‐impregnated polyurethane proves effective in killing *S. aureus* and *E. coli* within a short period of time which may have some applications in a clinical setting, however, its utility may be improved by chemical modification of the biocide to improve its stability.

## Conflict of Interest

No conflict of interest declared.

## Supporting information


**Data S1.** Microbiological protocol.
**Figure S1.** ATR spectrum of solvent‐treated polyurethane (control).
**Figure S2.** ATR spectrum of glutaraldehyde‐impregnated polyurethane.
**Figure S3.** ATR spectrum of glutaraldehyde‐coated polyurethane.
**Figure S4.** Carbon 1s region XPS spectrum for glutaraldehyde‐impregnated polyurethane surface.
**Figure S5.** Oxygen 1s region XPS spectrum for glutaraldehyde‐impregnated polyurethane surface.
**Figure S6.** Viable counts of (A) *S. aureus* for 1 h and (B) *E. coli* for 2 h after incubation at 20°C on modified polyurethane squares left for 30 days. Control samples are solvent treated.
**Table S1.** Average contact angle measurements (^o^) ± standard deviation, of water on a range of polyurethane polymer: untreated, solvent‐treated (control), glutaraldehyde‐impregnated, and glutaraldehyde‐coated samples after 30 days.
