# Supplementary material for: The bactericidal activity of glutaraldehyde‐impregnated polyurethane
Source: Microbiologyopen. 2016 Jun 3;5(5):891–7. doi: 10.1002/mbo3.378 (PMC5061724; doi:10.1002/mbo3.378)
Supplement: Supplementary file 1 — Data S1. Microbiological protocol. Figure S1. ATR spectrum of solvent‐treated polyurethane (control). Figure S2. ATR spectrum of glutaraldehyde‐impregnated polyurethane. Figure S3. ATR spectrum of glutaraldehyde‐coated polyurethane. Figure S4. Carbon 1s region XPS spectrum for glutaraldehyde‐impregnated polyurethane surface. Figure S5. Oxygen 1s region XPS spectrum for glutaraldehyde‐impregnated polyurethane surface. Figure S6. Viable counts of (A) S. aureus for 1 h and (B) E. coli for 2 h after incubation at 20°C on modified polyurethane squares left for 30 days. Control samples are solvent treated. Table S1. Average contact angle measurements (o) ± standard deviation, of water on a range of polyurethane polymer: untreated, solvent‐treated (control), glutaraldehyde‐impregnated, and glutaraldehyde‐coated samples after 30 days. [file MBO3-5-891-s001.docx]

**The bactericidal activity of glutaraldehyde-impregnated polyurethane**

**Sehmi S.,*^a,b,c^* Allan E.,*^c^* MacRobert A.J.*^b^* and Parkin I.P.**^a^***

*^a^* Materials Chemistry Research Centre, Department of Chemistry, University College London, 20 Gordon Street, London, WC1H 0AJ, UK. E-mail: i.p.parkin@ucl.ac.uk

*^b^* UCL Division of Surgery and Interventional Science, University College London, 67-73 Riding House Street, London, W1 W7EJ, UK.

*^c^* Division of Microbial Disease, UCL Eastman Dental Institute, University College London, 256 Gray’s Inn Road, London, WC1X 8LD, UK.

**Supporting Information**


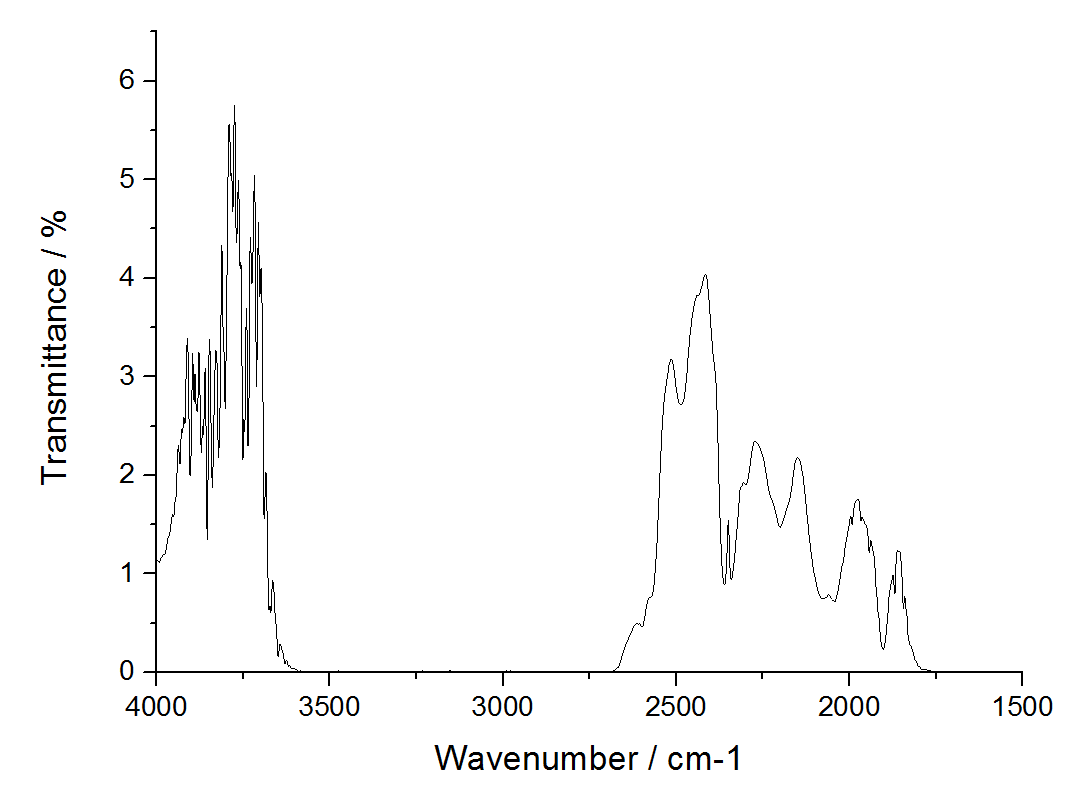


**Figure S1** ATR spectrum of solvent treated polyurethane (control).


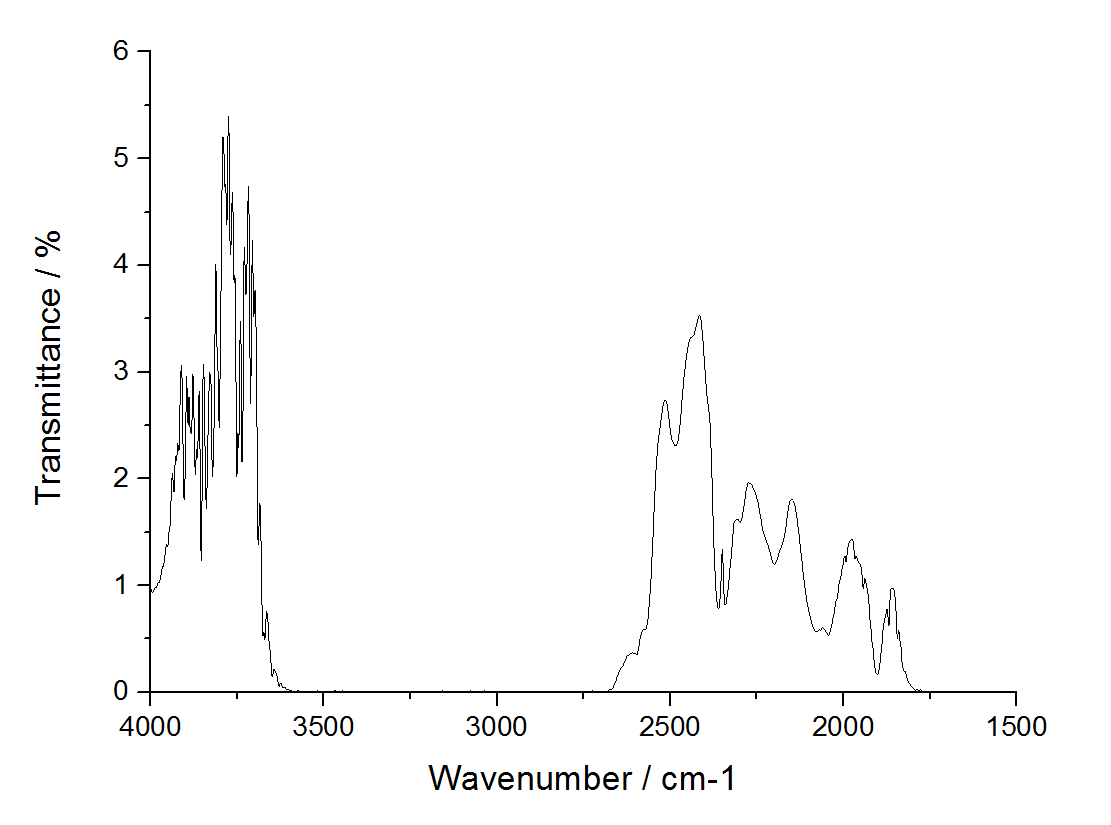


**Figure S2** ATR spectrum of glutaraldehyde-impregnated polyurethane.


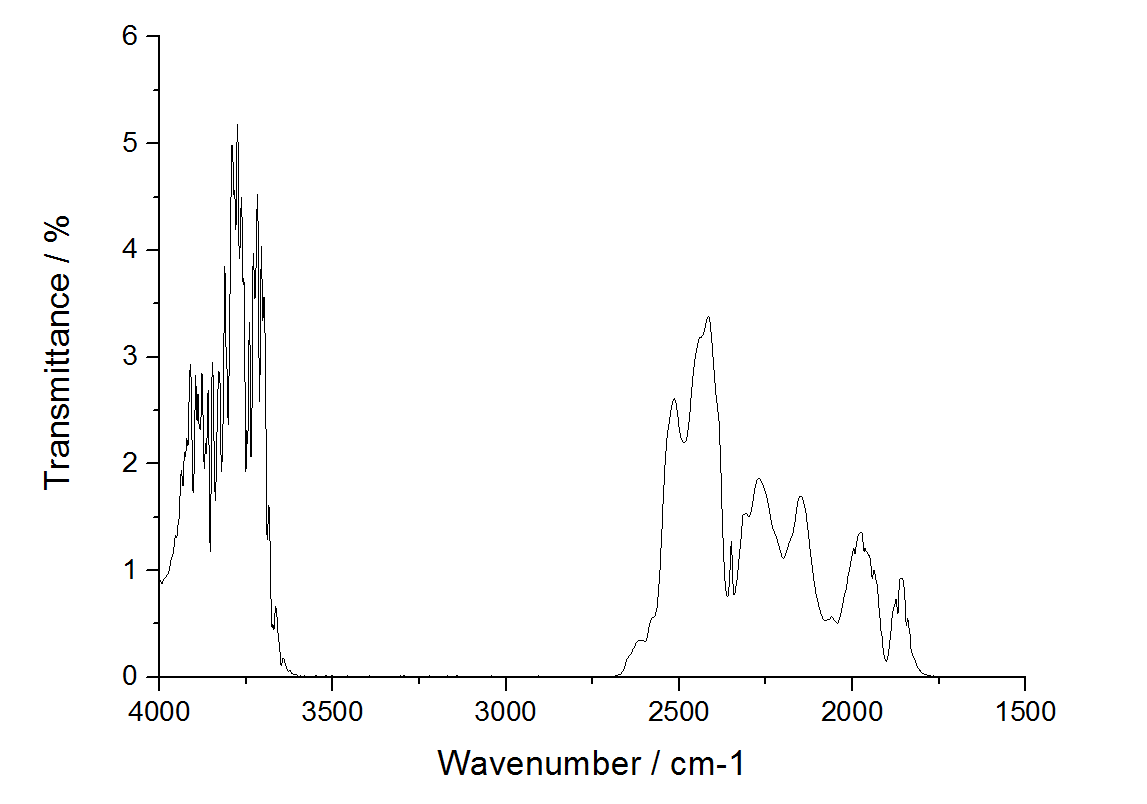


**Figure S3** ATR spectrum of glutaraldehyde-exposed polyurethane.

**Figure S4** Carbon 1s region XPS spectrum for glutaraldehyde-impregnated polyurethane surface.

**Figure S5** Oxygen 1s region XPS spectrum for glutaraldehyde-impregnated polyurethane surface.

**(a)**

**(b)**

**Figure S6** Viable counts of (a) *S. aureus* for 1 hour and (b) *E. coli* for 2 hours after incubation at 20^o^C on modified polyurethane squares left for 15 days. Control samples are solvent treated.

**Table S1** Average contact angle measurements (^o^) ± standard deviation, of water on a range of polyurethane polymer: untreated, solvent treated (control), glutaraldehyde-impregnated, and glutaraldehyde-coated samples after 30 days

| **Polymer sample** | **Contact angle (^o^)** |  | **Standard deviation** |
| --- | --- | --- | --- |
| Untreated | 92 | ± | 0.4 |
| Control | 94 | ± | 1.2 |
| Glutaraldehyde-impregnated | 94 | ± | 0.9 |
| Glutaraldehyde-coated | 93 | ± | 0.5 |

**Microbiological protocol**

BHI broth (10 mL) was inoculated with 1 bacterial colony and cultured in air (37 ^o^C, 200 rpm, 18 hours). The bacterial pellet was recovered by centrifugation, (20 ^o^C , 2867.2 *g*, 5 min), washed in PBS (10 mL) and centrifuged again to recover the bacteria (20 ^o^C, 2867.2 *g*, 5 min). The bacteria were finally re-suspended in PBS (10 mL). The washed bacterial suspension was diluted 1000-fold to achieve an inoculum of ~10^6^ cfu/mL. In each experiment, the inoculum was confirmed by plating 10-fold serial dilutions on agar for viable counts. Triplicates of each polymer sample type were inoculated with 25 µL of the inoculum and covered with a sterile cover slip (2.2 cm^2^). The samples were incubated for up to 2 hours in the dark. After incubation, the inoculated samples and cover slips were added to PBS (450 µL) and mixed thoroughly using a vortex mixer. The neat suspension and 10-fold serial dilutions were plated on agar for viable counts and incubated aerobically at 37 ^○^C for 24 (*E. coli*) or 48 hours (*S. aureus*).
